# Strategies to Enhance Protein Delivery

**DOI:** 10.1021/acs.langmuir.4c04636

**Published:** 2025-03-07

**Authors:** Yucheng Zhu, Weisi Zhuang, Hao Cheng

**Affiliations:** Department of Materials Science and Engineering, Drexel University, Philadelphia, Pennsylvania 19104, United States

## Abstract

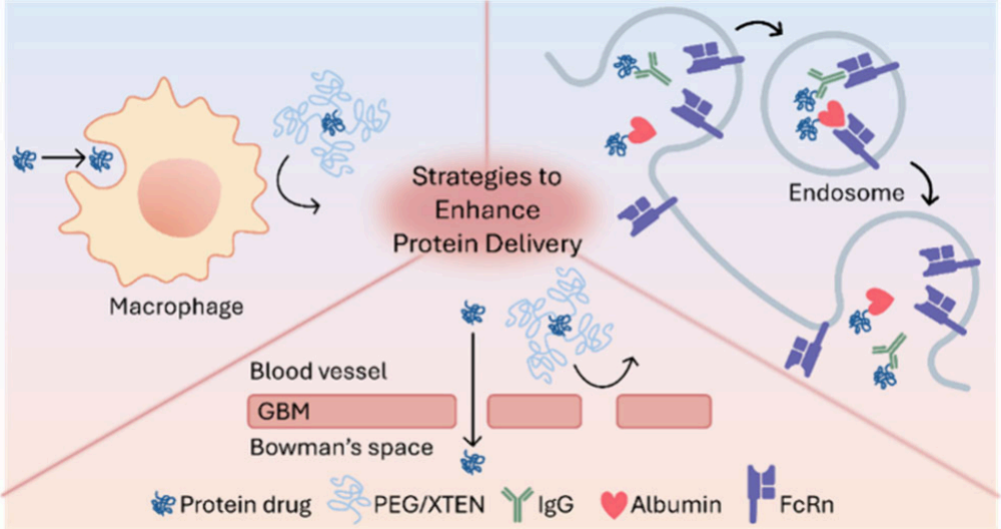

Therapeutic proteins play a crucial role in modern healthcare. However, the rapid clearance of proteins in the circulation system poses a significant threat to their therapeutic efficacy. The generation of anti-drug antibodies expedites drug clearance, resulting in another challenge to overcome in protein delivery. Several methods to increase the circulation half-lives of these proteins and to minimize their immunogenicity have been developed. This Review discusses the causes of protein clearance in the body, evaluates the FDA-approved strategies to prolong protein circulation, and highlights recent progress in the field. Additionally, the strengths and drawbacks of these methods and our perspectives for advancing protein delivery are provided.

## 1 Introduction

There has been unprecedented development of therapeutic proteins in the past three decades. More than 200 proteins have been approved by the FDA for treating various diseases, including hemophilia A and B,^1^ cancers,^2^ diabetes,^3^ growth hormone deficiency,^4^ autoimmune diseases,^5^ chronic inflammatory diseases,^6^ etc. However, the efficacy of therapeutic proteins is sometimes limited by their short circulation in the blood. This short circulation necessitates repeated dosing and high treatment concentrations, as maintaining certain protein levels is indispensable for effective treatment. In addition, therapeutic proteins are often recognized as “foreign” by the immune system, inducing the production of anti-drug antibodies in patients. The presence of these antibodies expedites the clearance of therapeutic proteins from the human body. The short circulation half-life of the proteins and their immune responses not only reduce their therapeutic efficacy but may also cause side effects, for example, hypersensitivity reactions.^7^

Owing to extensive research, various strategies to extend the circulation half-lives of therapeutic proteins have been developed. The FDA-approved strategies, including PEGylation,^8^ XTENylation,^9^ Fc fusion,^10^ and albumin attachment,^11^ are the focus of this Review, along with other emerging approaches under development.

## 2 Mechanisms of Protein Clearance in the Body

**Figure 1:**
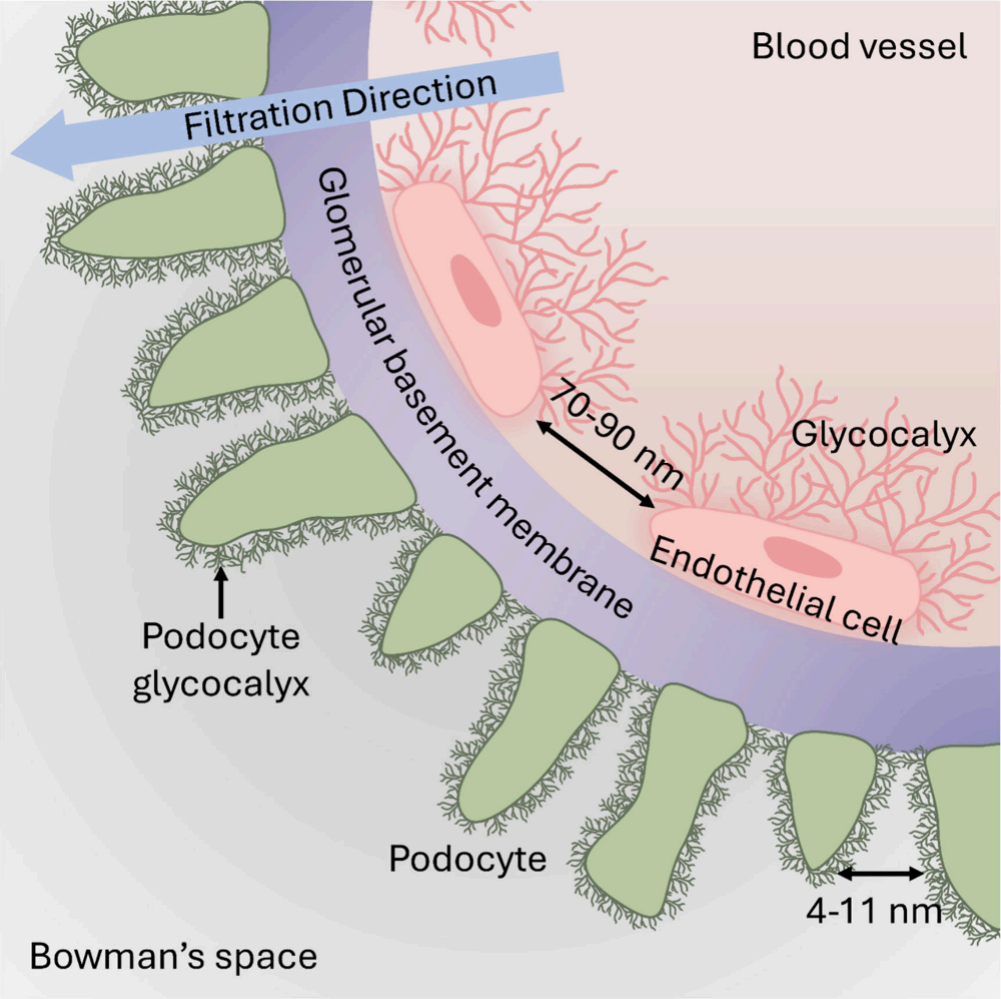
Protein clearance in the kidneys by filtration membranes under increased blood pressure. The membranes are composed of an endothelial cell layer, a glomerular basement membrane (GBM), and podocytes.

The human body has three main mechanisms to clear therapeutic proteins. The first mechanism is renal clearance. Blood pressure is increased in the kidneys due to the narrow efferent arterioles. The high blood pressure pushes the blood through filtration membranes, which are composed of an endothelial cell layer, a glomerular basement membrane (GBM), and podocytes.^12^ While the filtered blood returns to circulation, the filtration membrane allows proteins with a hydrodynamic size of smaller than 6 nm to pass through, clearing them into the urine (Figure 1). Larger proteins mostly remain in the blood.

**Figure 2:**
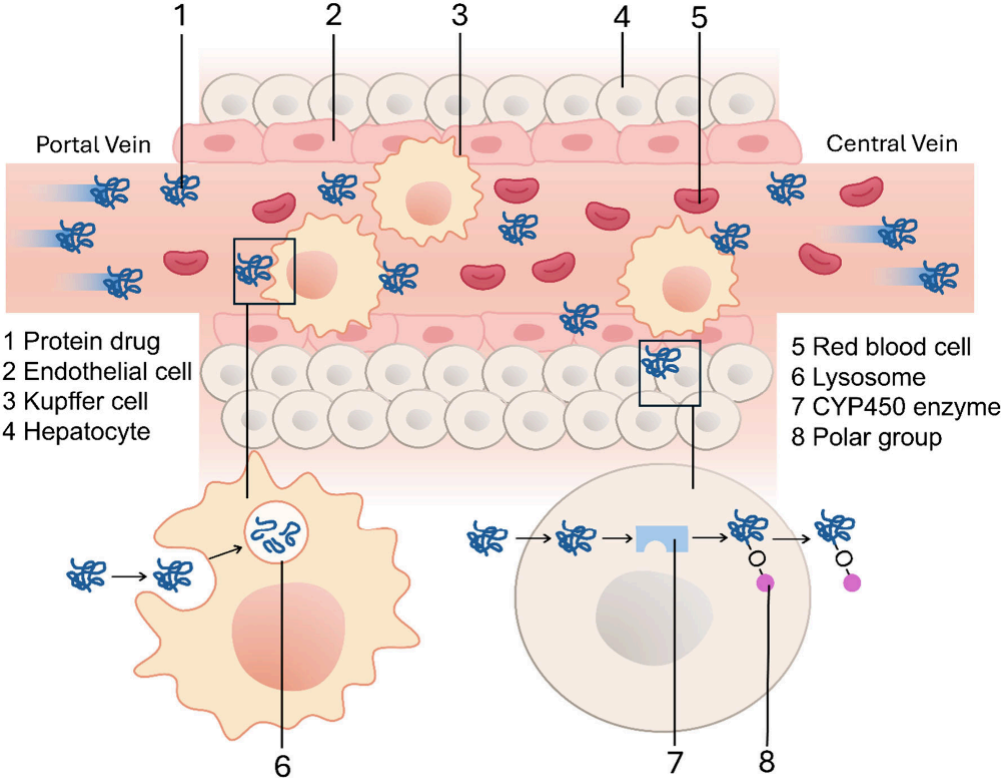
Protein clearance in the liver by Kupffer cells and hepatocytes.

Metabolism is another key mechanism of protein clearance. Metabolism occurs in various organs such as kidneys and the gastrointestinal tract but primarily in the liver. Metabolic rates depend on many factors, including the molecular weight, secondary and tertiary structures, and glycosylation levels of proteins. Most protein metabolism occurs inside hepatocytes in the liver. Small peptides consisting of fewer than 10 amino acids with high hydrophobicity can pass through hepatocyte membranes via passive diffusion,^13^ whereas larger peptides or proteins are mostly internalized by hepatocytes through the mediation of receptors on the hepatocyte surface. These receptors can be either nonspecific, like low-density lipoprotein receptor-related protein, or specific, like the Fc-γ receptor. Once internalized, proteins are metabolized by heme-containing enzymes in the cytochrome P450 system (Cyp450), located in either the smooth endoplasmic reticulum or mitochondria. The Cyp450 system is a family of enzymes that are responsible for the metabolism of most drugs. It can bind proteins and introduce polar groups such as hydroxyl and thiol groups through oxidation, reduction, and/or hydrolysis. The modified proteins are then conjugated to polar compounds catalyzed by transferase enzymes. The proteins with polar compounds are recognized by efflux transporters and pumped out of cells.^14^ Then, the proteins enter the kidney or bile system and be cleared into urine or feces.^15^ Apart from metabolism, proteins can also be cleared by Kupffer cells and liver sinusoidal endothelial cells in the liver by the capture and degradation in the lysosome (Figure 2).

The last mechanism is enzymatic degradation, which can occur in any organ within the body. It can be site-specific or nonspecific and intracellular or extracellular. Enzymatic degradation is influenced by the hydrodynamic size and secondary and tertiary structure of the protein. For example, the d configuration of amino acids is more stable than other configurations as few enzymes can hydrolyze the peptide bonds in d-configured amino acids. Besides, β-amino acids typically form more stable amide bonds to resist cleavage by peptidase.^16^

## 3 Strategies to Extend the Circulation Half-Life of a Protein

### 3.1 PEGylation

**Figure 3:**
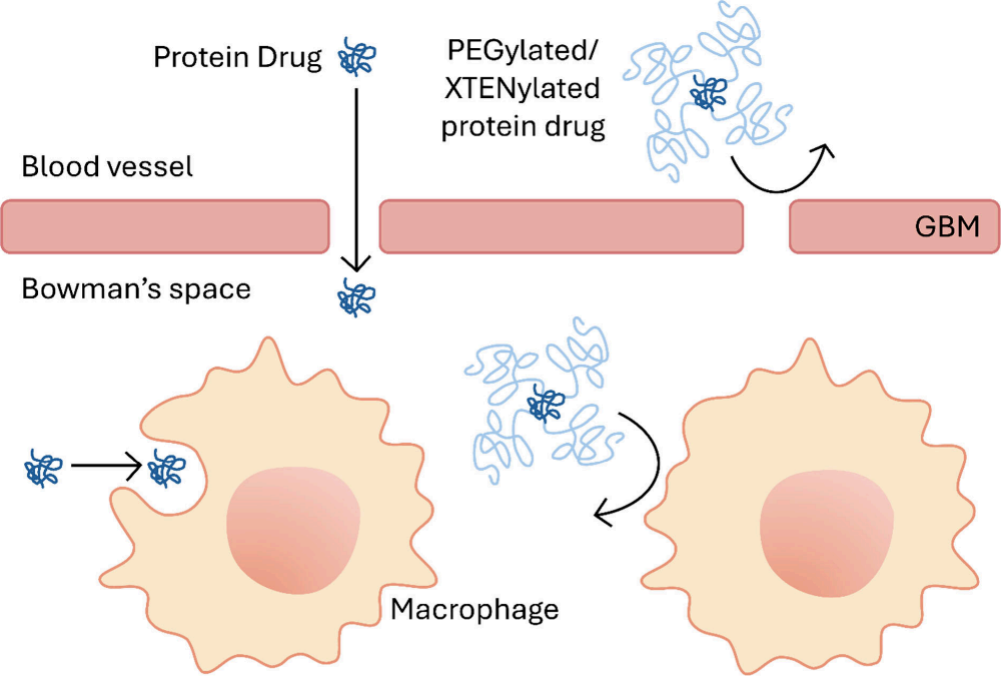
PEGylation and XTENylation reduce renal clearance and uptake by macrophages.

PEGylation refers to conjugation of poly(ethylene glycol) (PEG) chains to therapeutics or the generation of PEG layers around nanomedicines. Because of its excellent biocompatibility and relatively low immunogenicity, PEG has been approved by the FDA as an ingredient in medicine. PEG is less immunogenic than many therapeutics, for example, proteins derived from animals or produced by bacteria. However, when PEGs are linked to immunogenic proteins or nanomedicines, anti-PEG immune responses can be elicited.^17^ As of 2024, 38 PEGylated drugs, including lipids, peptides, proteins (Table 1), and nanoparticles, have been approved by the FDA.^18^ PEG extends the circulation half-lives of proteins via two main mechanisms. First, hydrophilic PEG forms a hydration layer around the protein to increase its hydrodynamic size, resulting in reduced renal clearance. Second, PEG provides a stealth effect, shielding the proteins from receptor-mediated internalization and enzymatic degradation. The binding of opsonins, including antibodies and complement proteins, to therapeutic proteins and nanomedicines results in the clearance of therapeutics by immune cells.^19^ The flexible PEG chain, characterized by its C–O–C backbone, interferes with the binding of protected proteins to plasma proteins and immune cell receptors through thermodynamically driven entropic repulsion. This repulsion arises because such interactions confine the flexible PEG chain, reducing its entropy and making the process thermodynamically unfavorable (Figure 3).^20^

**Table 1:** FDA-Approved PEGylated Protein Drugs

generic name	brand name	approval year	description	half-life in humans	application	ref
pegademase bovine	Adagen	1990	PEGylated enzyme adenosine deaminase	3–6 days	severe combined immunodeficiency disease	(24)
pegaspargase	Oncaspar	1994	PEGylated l-asparaginase	5.8 days	acute lymphoblastic leukemia	(25)
peginterferon alfa-2b	Pegintron	2001	PEGylated alfa-2b	40 h	chronic hepatitis C	(26)
peginterferon alfa-2a	Pegasys	2002	PEGylated alfa-2a	160 h	chronic hepatitis B and C	(27)
pegfilgrastim	Neulasta	2002	PEGylated G-CSF	15–80 h	stimulation of white cell production	(28)
pegvisomant	Somavert	2003	PEGylated growth hormone	74–172 h	acromegaly	(29)
pegaptanib	Macugen	2004	PEGylated aptamer	10 days	neovascular age-related macular degeneration	(30)
certolizumab pegol	Cimzia	2008	PEGylated TNF blocker	14 days	rheumatoid arthritis and Crohn’s disease	(31)
methoxy polyethylene glycol-epoetin β	Mircera	2007	PEGylated erythropoietin	119 h	anemia associated with chronic kidney disease	(32)
pegloticase	Krystexxa	2010	PEGylated uricase	300 h	chronic gout	(33)
peginterferon alfa-2b	Sylatron	2011	PEGylated interferon alfa-2b	51 h	melanoma	(34)
peginterferon β-1a	Plegridy	2014	PEGylated interferon β-1a	78 h	multiple sclerosis	(35)
antihemophilic factor (recombinant), PEGylated	Adynovate	2015	PEGylated FVIII	13.4–14.7 h	hemophilia A	(36)
coagulation factor IX (recombinant), glycoPEGylated	Rebinyn	2017	PEGylated FIX	114.9 h	hemophilia B	(37)
pegvaliase-pqpz	Palynziq	2018	PEGylated phenylalanine ammonia lyase	47 h	phenylketonuria	(38)
ropeginterferon alfa-2b	Besremi	2021	PEGylated interferon	7 days	polycythemia vera	(39)
pegcetacoplan	Empaveli	2021	PEGylated pentadecapeptide	8 days	paroxysmal nocturnal hemoglobinuria	(40)
avacincaptad pegol	Izervay	2023	PEGylated ribonucleic acid aptamer	12 days	geographic atrophy	(41)
pegunigalsidase alfa-iwxj	Elfabrio	2023	PEGylated human GLA enzyme	96.5 h	Fabry disease	(42)
palopegteriparatide	Yorvipath	2024	PEGylated parathyroid hormone	60 h	hypoparathyroidism	(43)

PEGylated therapeutic proteins have been used to treat various diseases, including hepatitis C, acromegaly, leukemia, anemia, and neutropenia.^8^ The first PEGylated protein drug, pegademase bovine, was approved by the FDA in 1990. It is used to treat severe combined immunodeficiency disease (SCID) caused by a deficiency in adenosine deaminase (ADA).^21^ Native ADA has a circulation half-life of 30 min in mice after intravenous (*i.v.*) injection. After conjugation of 5 kDa PEG chains, the circulation half-life in mice increased to 28 h.^22^ In humans, the circulation half-life of PEGylated ADA was shown to range from 3 to 6 days.^21^ There were no serious adverse effects reported over several years, demonstrating the safety of PEGylated ADA.^23^

Uricase is an enzyme that terminates purine catabolism, converting weakly water-soluble uric acid into more soluble allantoin.^44^ Since humans cannot synthesize uricase, the buildup of uric acid in joints causes pain and swelling, a disease known as gout. Animal-derived uricase has been used to treat gout. However, the immunogenicity of the protein leads to the production of anti-uricase antibodies upon frequent dosing, thereby decreasing the efficacy of uricase. In 2005, Ganson et al. developed PEGylated uricase and tested its efficacy in patients with gout.^45^ They found that the terminal half-life of subcutaneously injected PEGylated uricase was between 10 and 20 days, which is approximately 13–26 times longer than that of native uricase. The plasma urate concentration decreased with increasing doses of PEGylated uricase. Despite a subset of patients exhibiting low levels of anti-uricase antibodies, PEGylated uricase is mostly safe. In 2010, the FDA approved a PEGylated uricase for treating refractory chronic gout.^46^

Repeated injections of PEGylated immunogenic proteins induce immune responses against PEG in some patients, generating anti-PEG antibodies.^47^ These antibodies have a high binding affinity for the PEG backbone and end groups. Once bound, these antibodies can enhance the recognition and uptake of PEGylated proteins by immune cells and induce activation of the complement system, potentially triggering inflammatory responses and adverse effects, such as nasal pruritus, conjunctivitis, and dizziness. An increasing number of people have developed preexisting anti-PEG antibodies due to frequent exposure to PEG in cosmetics and food additives, complicating the use of PEGylated medicines.^47^ Furthermore, PEG metabolism by enzymes such as those of the P450 family is relatively minimal and slow due to the nondegradability of PEG.^48^ The clearance of PEG by the kidney is limited to PEG with a molecular weight of less than 30 kDa. PEG with a molecular weight between 30 and 50 kDa is internalized by liver parenchymal cells and released into the bile via exocytosis before being excreted from the body through feces. PEG with a molecular weight beyond 50 kDa is stored in Kupffer cells, potentially leading to the formation of vacuoles.^49^ The accumulation of PEG in these vacuoles may increase the risk of toxicity.^50^ Therefore, high-molecular weight PEG is not used in PEGylation to avoid potential toxicity. Branched PEG that can degrade into low-molecular weight PEG has been used to minimize the accumulation of PEG in the body. For instance, branched PEG is used in ADYNOVATE, a drug for hemophilia A treatment.^51^

### 3.2 XTENylation

XTEN is a class of non-immunogenic polypeptides composed of hydrophilic amino acids A, E, G, P, S, and T.^9^ XTEN polypeptides lack secondary structures and therefore are hydrodynamically larger than proteins with similar molecular weights. XTEN can be genetically fused to a therapeutic protein at specific sites to minimize the impact of bulky XTEN on the bioactivity of the proteins. It can also be chemically conjugated to protein drugs through functional groups such as amine and thiol. After being attached to proteins, XTEN extends the protein circulation time by reducing renal clearance due to the increased hydrodynamic size. Besides, the long polypeptide chains can hinder internalization by immune cells (Figure 3). Since its introduction in 2009, XTEN has been considered an alternative to PEG owing to its low immunogenicity and biodegradability. In February 2023, Altuviiio became the first XTENylated drug approved by the FDA for treating hemophilia A in both adults and children^52^ (Table 2).

**Table 2:** FDA-Approved XTENylated Drugs

generic name	brand name	approval year	description	half-life in humans	application	ref
Efanesoctocog alfa	Altuviiio	2023	XTENylated FVIII	48.2 h	hemophilia A	(52)

**Figure 4:**
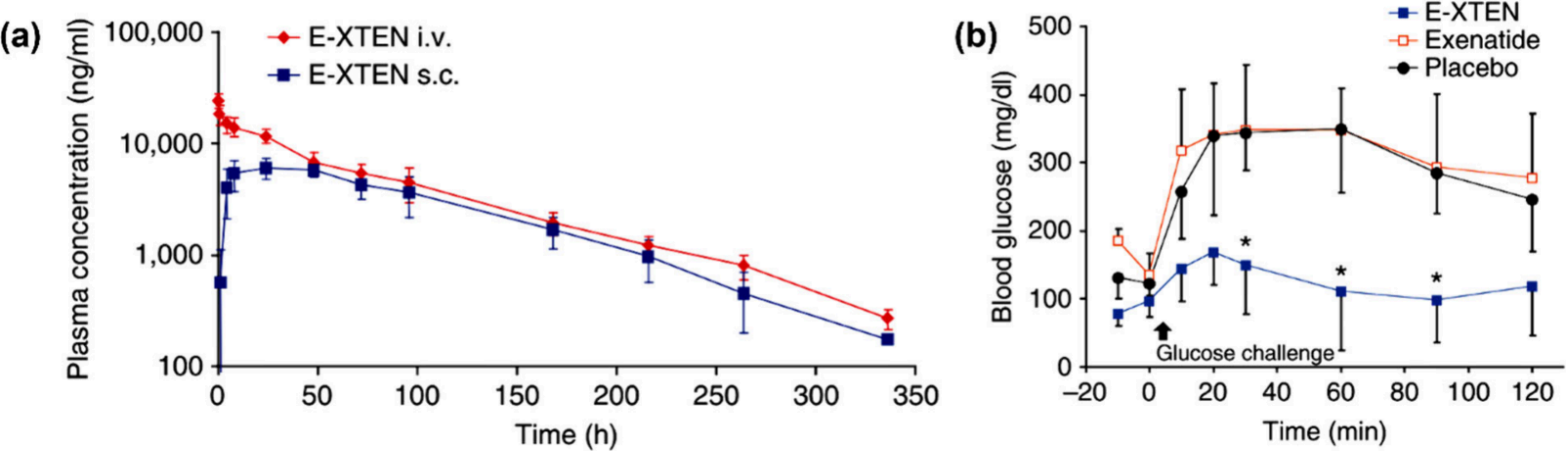
Pharmacokinetics and efficacy of E-XTEN in mice. (a) Pharmacokinetic plasma profile of E-XTEN dosed at 1 mg/kg either intravenously (iv) or subcutaneously (sc) in cynomolgus monkeys. (b) *In vivo* efficacy of E-XTEN in mice. Reproduced with permission from ref (3). Copyright 2009 Springer Nature.

According to a report from the Centers for Disease Control and Prevention (CDC), about 1 in 10 Americans developed diabetes in 2021. Exenatide, a 39-amino acid peptide agonist of the glucagon-like peptide-1 (GLP-1) receptor, is widely used for combating type 2 diabetes. However, due to rapid renal clearance, the circulation half-life of exenatide is only around 2.4 h.^3^ For treatment of type 2 diabetes, injections must be administered twice daily, posing a significant burden for patients. In 2009, Schellenberger et al. developed an 864-amino acid XTEN-fused exenatide.^3^ After purification, the homogeneous product was achieved and retained chiral properties similar to those of native exenatide. The circulation profile of this fused protein was first tested in monkeys, where the circulation half-life was determined to be 60 h through iv injection (Figure 4a). Using allometric scaling, the projected circulation half-life in humans would be 139 h, approximately 60 times longer than that of native exenatide. The group also tested XTEN-fused exenatide by using subcutaneous injection and achieved approximately 80% bioavailability. The maximum plasma concentration was reached after 24–48 h, with plasma levels remaining nearly linear before entering the elimination phase. A mouse glucose challenge model was used to test the efficacy of the drug. It was shown that the XTEN-fused exenatide effectively resisted the glucose challenge for up to 48 h, whereas native exenatide had no effect (Figure 4b).

In addition to reducing renal clearance, XTEN also enhances protein delivery by reducing receptor-mediated clearance. Human growth hormone (HGH) therapy for patients with growth hormone deficiency requires daily injections for several years. HGH is cleared by both renal and receptor-mediated mechanisms, resulting in a short circulation half-life of around 2.4 h in monkeys.^53^ Growth hormone receptors are widely distributed among different organs, with the highest concentration on the surface of liver cells.^54^ In 2012, Cleland et al. developed a XTEN-fused human growth hormone to simultaneously reduce both renal and receptor-mediated clearance.^55^ XTEN fusion at the N-terminus alone reduced renal clearance and increased the circulation half-life to 49 h in monkeys. Fusion at the N- and C-termini reduced renal and receptor-mediated clearance, further prolonging the circulation half-life to 110 h in monkeys, which is 46 times longer than that of native HGH.

The major disadvantages of XTEN lie in the fact that it must be a huge molecule to achieve long blood circulation of fusion proteins. The large hydrodynamic size of XTEN alters the biodistribution of the fused peptides and proteins by preventing their diffusion in tissues and reducing their bioactivity due to the blockage of their binding to targets.^56^ In addition, XTENylation significantly increases the manufacturing cost. Although XTEN has shown low immunogenicity in reports, further studies of its immunogenicity are needed because it has not been used as broadly as PEG.

### 3.3 Fc Fusion

**Figure 5:**
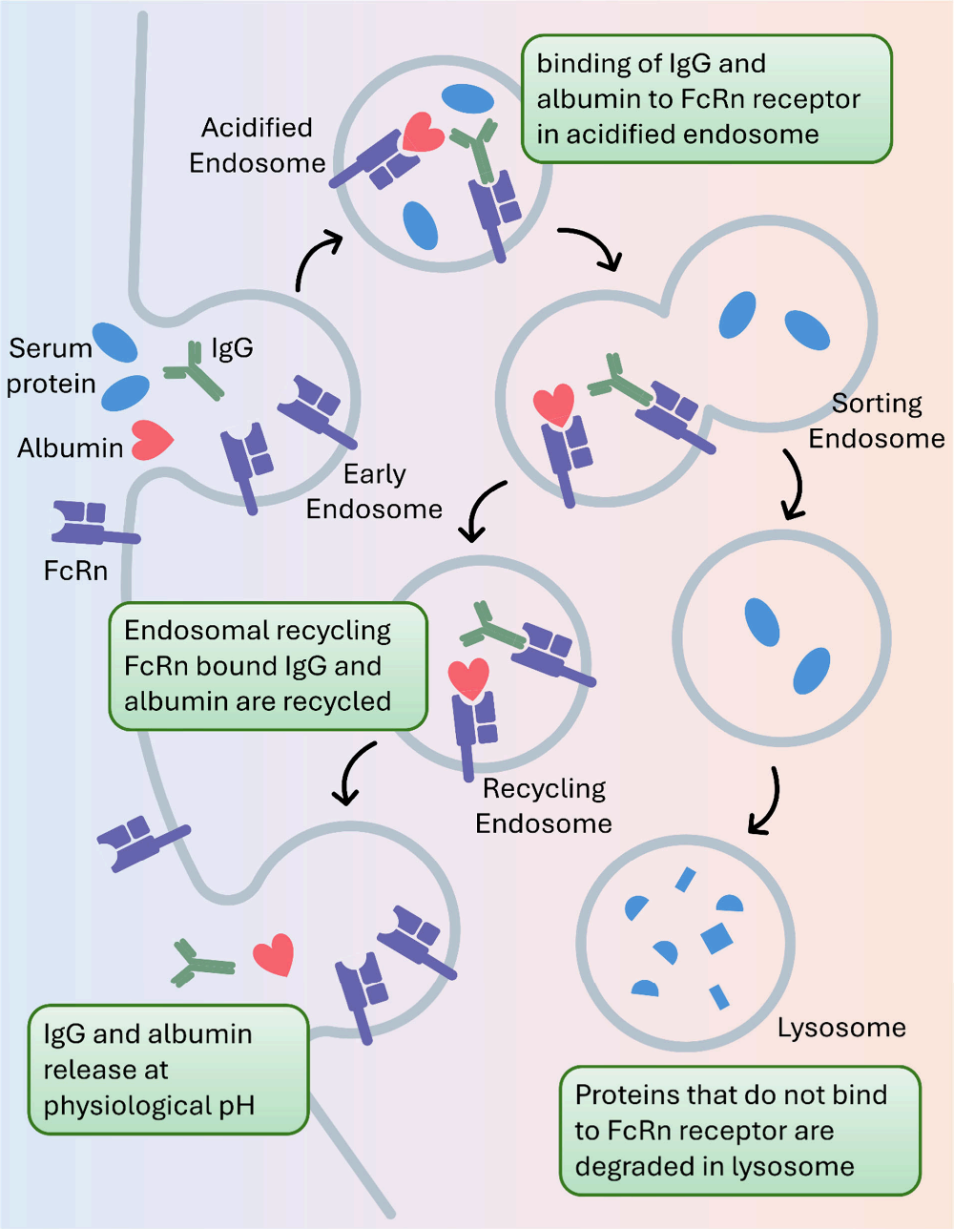
FcRn-mediated recycling of IgG and albumin.

Some endogenous proteins like immunoglobulin G (IgG) have a circulation half-life of 2–3 weeks.^57^ The long circulation half-life is achieved through the neonatal Fc receptor (FcRn)-mediated recycling mechanism.^58^ The Fc region of the tail part of IgG consists of two heavy chains. After internalization by immune cells through endocytosis, the Fc region strongly binds to FcRn in immune cells under the acidic conditions of the early endosome. FcRn is capable of transporting IgG across cell monolayers either from basolateral to apical or from apical to basolateral.^59^ The FcRn–IgG complex is sorted to a common recycling endosome, whereas all unbound proteins are directed to lysosome and degraded there. The recycling endosome arrives at the cell surface and fuses with the cell membrane, where the local pH changes to physiological pH.^60^ At physiological pH, IgG dissociates from FcRn and reenters the bloodstream (Figure 5). It was postulated that after fusion with the Fc region proteins could be recycled in a manner similar to that of IgG. More than a dozen Fc-fused protein drugs have been approved by the FDA (Table 3).

**Table 3:** FDA-Approved Fc-Fused Drugs

generic name	brand name	approval year	description	half-life in humans	application	ref
etanercept	Enbrel	1998	Fc-fused TNF receptor	102 h	autoimmune diseases	(61)
abatacept	Orencia	2005	Fc-fused CTLA4	16.7 h	rheumatoid arthritis	(62)
rilonacept	Arcalyst	2008	Fc-fused IL-1 antagonist	8.6 days	cryopyrin-associated periodic syndromes	(63), (64)
romiplostim	Nplate	2008	Fc-fused thrombopoietin receptor agonist	3.5 days	thrombocytopenia in immune thrombocytopenia	(65)
belatacept	Nulojix	2011	Fc-fused CTLA4	9.8 days	prevention of organ rejection	(66)
aflibercept	Eylea	2011	Fc-fused VEGF receptor	5–6 days	neovascular age-related macular degeneration	(67)
aflibercept	Zaltrap	2012	Fc-fused VEGF receptor	6 days	metastatic colorectal cancer	(68)
coagulation factor IX (recombinant), Fc fusion protein	Alprolix	2014	Fc-fused FIX	86 h	hemophilia B	(69)
efmoroctocog alfa	Eloctate	2014	Fc-fused FVIII	19.7 h	hemophilia A	(70)
dulaglutide	Trulicity	2014	Fc-fused GLP-1 receptor agonist	5 days	type 2 diabetes mellitus	(71)
asfotase alfa	Strensiq	2015	Fc-fused asfotase alfa	5 days	hypophosphatasia	(72)
luspatercept-aamt	Reblozyl	2019	Fc-fused human activin receptor type IIB	11 days	anemia	(73)
efanesoctocog alfa	Altuviiio	2023	Fc-fused FVIII	48.2 h	hemophilia A	(52)
nogapendekin alfa inbakicept	Anktiva	2024	Fc-fused IL-15 receptor	N/A	non-muscle invasive bladder cancer	(74)

The FDA approved the first Fc fusion protein, etanercep, which is an Fc-fused tumor necrosis factor (TNF) receptor, in 1998. TNF-α is an inflammation cytokine that plays a pivotal role in autoimmune diseases, for example, rheumatoid arthritis (RA). The TNF receptor has been shown to alleviate the symptoms of autoimmune diseases by inhibiting TNF-α. However, the native TNF receptor has a short circulation half-life ranging from 20 to 30 h.^75^ After fusion with Fc, the circulation half-life is prolonged to around 100 h.^76^ Etanercep has demonstrated a good therapeutic efficacy against RA with weekly injections.^77^ In a human–endotoxin challenge model, etanercep has been shown to effectively neutralize TNF-α induced by endotoxin.^76^

As another example, patients with hemophilia A or B experience prolonged blood clotting times due to genetic disorders. Coagulation factor VIII, which is administered every other day as a treatment for hemophilia A, was also fused with Fc (rFVIIIFc).^78^ This resulted in a 1.6-fold increase in circulation half-life with no adverse side effects or detection of anti-FVIII inhibitors. FDA-approved factor VIII from Sanofi, Altuviiio, leveraged both Fc fusion and XTEN to extend its circulation.^79^ The half-life is more than 40 h, enabling once-weekly administration.^80^

**Figure 6:**
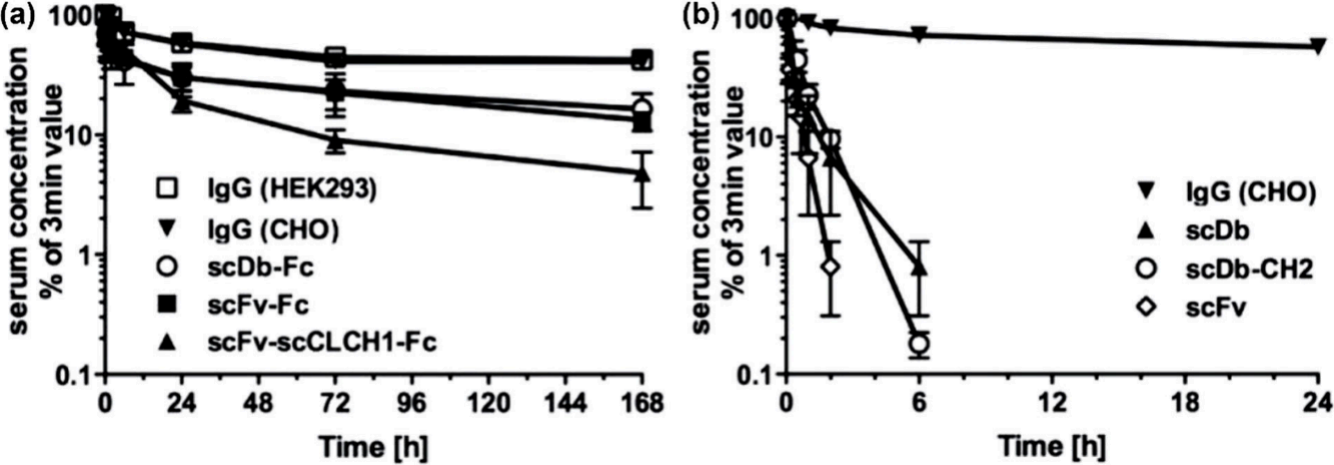
Comparison of the circulation times of IgG and Fc fusion proteins. (a) Pharmacokinetics of IgG and Fc fusion proteins. (b) Pharmacokinetics of scFv, scDb, and scFv-scCLCH1-Fc. Reproduced with permission from ref (10). Copyright 2016 Taylor & Francis Group, LLC.

Theoretically, Fc-fused proteins should have a circulation half-life similar to that of IgG. In 2016, Unverdorben et al. fused Fc with several carcinoembryonic antigen-targeted proteins such as single-chain variable fragments (scFv), single-chain diabody (scDb), and scFv fused to a single-chain CL-CH1 (scCLCH1).^10^ After fusion, these proteins had molecular weights similar to those of IgG. Following iv injection into mice, all fused proteins exhibited a dramatic increase in circulation half-life compared to those of their native counterparts. Nevertheless, their circulation half-lives were significantly shorter than that of IgG (Figure 6a,b). This phenomenon may be explained by variations in the binding affinity of Fc against FcRn, potentially due to factors such as the protein structure, the connecting arms of the Fc region, steric hindrance, and glycosylation. Overall, the rate of dissociation between the Fc region of IgG and FcRn may be faster than that of fusion proteins, resulting in less IgG retention.

Despite its clear advantage in extending protein circulation in the blood, Fc fusion technology suffers from some notable disadvantages. First, the Fc region may interact with Fc receptors on immune cells and complement proteins, triggering antibody-dependent cell-mediated cytotoxicity or complement-dependent cytotoxicity.^81^ Second, this approach cannot be applied to protein drugs that function intracellularly, for example, the p53 tumor suppressor protein.^82^ Third, the large size of Fc reduces the rate of diffusion of the fused proteins. In addition, this approach increases manufacturing costs, lacks the flexibility to control pharmacokinetics, and may destabilize proteins due to incorrect folding.^83^

### 3.4 Albumin Attachment

Albumin is another endogenous protein with a long circulation half-life, achieved through FcRn-mediated recycling^58^ (Figure 5). After internalization, albumin binds to FcRn in acidic endosomes and is released at physiological pH through exocytosis. Notably, although albumin and IgG are both recycled through binding to FcRn, they do not interfere with each other in binding. There are three methods to attach albumin to a therapeutic protein. The first method involves using a helper molecule to noncovalently bind albumin. For example, insulin detemir is insulin conjugated with a fatty acid (myristic acid) at the amino acid lysine. In circulation, the fatty acid forms a noncovalent bond with endogenous albumin, which dissociates slowly.^84^ In chronic weight management, both semaglutide (GLP-1R agonist peptide) from Novo Nordisk^85^ and tirzepatide (GLP-1R/GIP-R dual agonist peptide) from Eli Lilly^86^ utilized a similar approach.^87^ Albumin can also be attached to therapeutic proteins through genetic fusion or chemical conjugation via a linker. The FDA-approved therapeutic proteins utilizing albumin for extended circulation are summarized in Table 4.

**Table 4:** FDA-Approved Albumin Modulators

generic name	brand name	approval year	description	half-life in humans	application	ref
nab-paclitaxel	Abraxane	2005	albumin-bound paclitaxel	13–27 h	breast cancer	(88)
insulin detemir	Levemir	2005	insulin detemir with a fatty acid chain that can bind to endogenous albumin	5–7 h	diabetes mellitus	(89)
albumin (human)	Flexbumin	2005	human albumin	15–20 days	hypovolemia	(90)
gadofosveset trisodium	Vasovist	2008	gadolinium-based MRI contrast agent that can bind to endogenous albumin	16.3 h	magnetic resonance angiography	(91)
liraglutide	Victoza	2010	lipidated GLP-1 recepor agonist that can bind to endogenous albumin	13 h	type 2 diabetes mellitus	(92)
albumin (human)	Kedbumin	2011	human albumin	19 days	hypoalbuminemia	(93)
coagulation factor IX (recombinant), albumin fusion protein	Idelvion	2016	albumin-fused FIX	104 h	hemophilia B	(94)
semaglutide	Ozempic	2017	GLP-1 receptor agonist with a fatty diacid moiety that can bind to endogenous albumin	1 week	type 2 diabetes mellitus	(95)
sirolimus protein-bound	Fyarro	2021	sirolimus formulated as albumin-bound nanoparticles	59 h	perivascular epithelioid cell tumor	(96)
tirzepatide	Zepbound	2022	GIP receptor and GLP-1 receptor agonist with a fatty diacid moiety that can bind to endogenous albumin	5 days	chronic weight management	(97)

**Figure 7:**
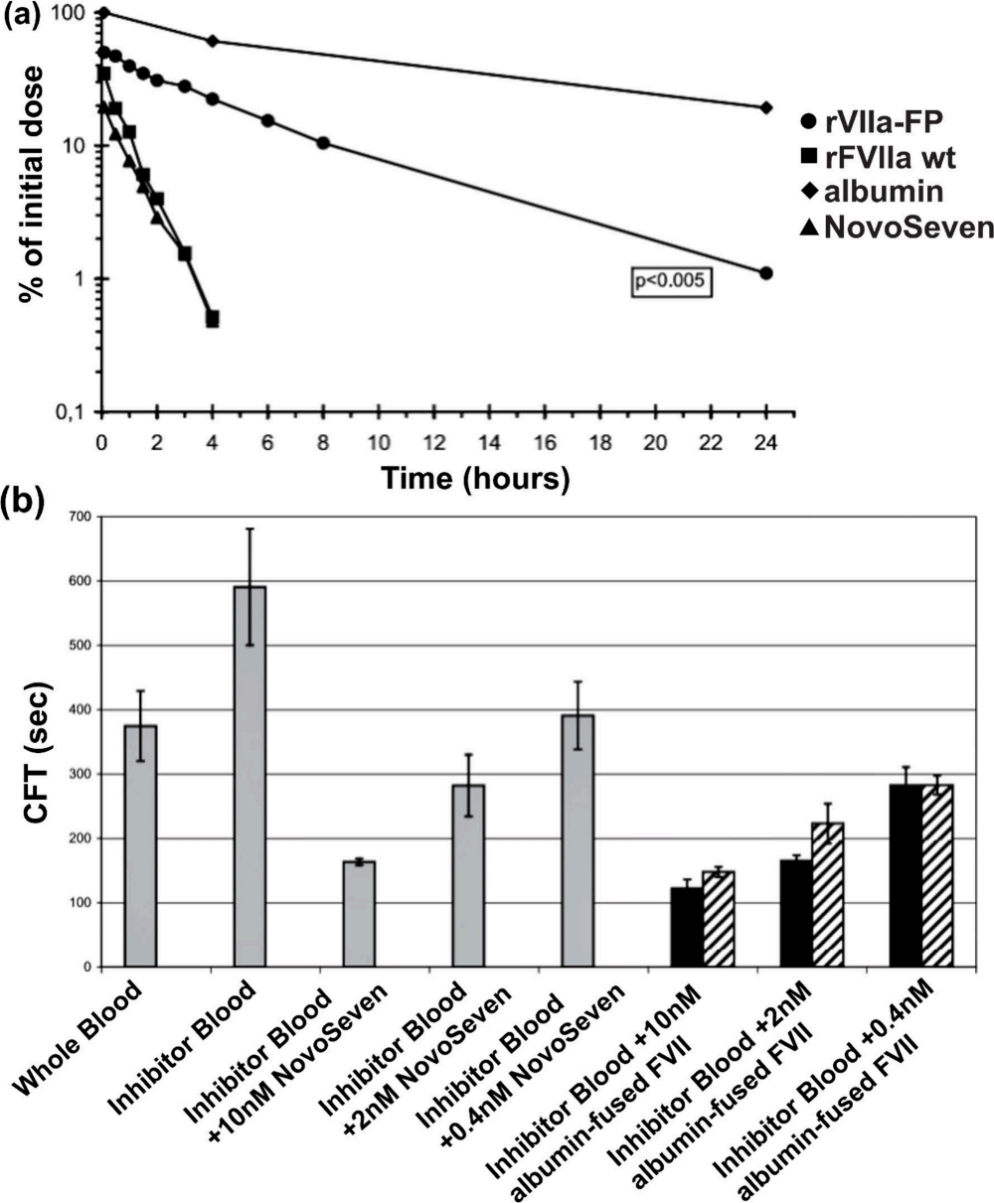
Blood circulation of FVII and clot formation time. (a) Circulation profile of different FVII in rats. (b) *In vitro* evaluation of albumin-fused FVII and NovoSeven in human inhibitor whole blood. Reproduced with permission from ref (99). Copyright 2008 Schattauer GmbH.

Coagulation factor VII (FVII) is another crucial protein involved in blood clotting. In 1996, recombinant activated FVII (FVIIa) became commercially available as a treatment option for both hemophilia A and B, known as NovoSeven.^98^ FVIIa has a very short half-life, necessitating frequent administration. In 2008, Weimer et al. genetically fused albumin onto FVIIa.^99^ The fused FVIIa showed a circulation half-life 5.8 and 6.7 times longer than those of recombinant FVIIa and NovoSeven, respectively (Figure 7a). However, compared to that of native albumin, fused FVIIa displayed a notably shorter circulation half-life, likely due to differences in their binding affinities with FcRn. The group conducted an experiment to test the clot formation time in blood with FVIII activity inhibitors. Both NovoSeven and albumin-fused FVIIa were able to decrease the clot formation time in a dose-dependent manner. Interestingly, albumin-fused FVIIa showed a clot formation time even shorter than that of NovoSeven (Figure 7b).

Compared to Fc fusion, the strategy of albumin attachment offers advantages, such as low immunogenicity, reduced manufacturing cost, and greater flexibility in controlling the pharmacokinetics of peptides and proteins by adjusting their binding with albumin. This approach is particularly suitable to extend the circulation of peptides. However, as in the cases of XTENylation and Fc fusion, the increased size due to the association with albumin limits the diffusion of therapeutics in tissues. Additionally, albumin may bind to other nontargeted cells through non-FcRn albumin receptors, like gp60,^100^ decreasing the efficacy of protein therapeutics.

### 3.5 Antibody-Conjugated or -Fused Cytokine

As orchestrators of the immune system, cytokines play a crucial role in cancer treatment.^101^ However, there are two major obstacles to their efficacy and safety: the short half-life of cytokines and the systemic toxicity caused by poor specificity in targeting. While conjugation with PEG or IgG can prolong blood circulation, researchers have also employed antibodies to enhance the targeting specificity of cytokines.

Some antibodies exhibit a high affinity for surface antigens on tumor cells. Upon binding, these antibodies can inhibit tumor growth or directly kill tumor cells through a number of mechanisms, including complement activation, antibody-dependent cellular toxicity, blockade of growth factors, antiproliferative effects, and pro-apoptotic effects.^102^ It was hypothesized that combining antibodies with cytokines may improve therapeutic efficacy by enhancing specificity, in addition to prolonging circulation.

**Figure 8:**
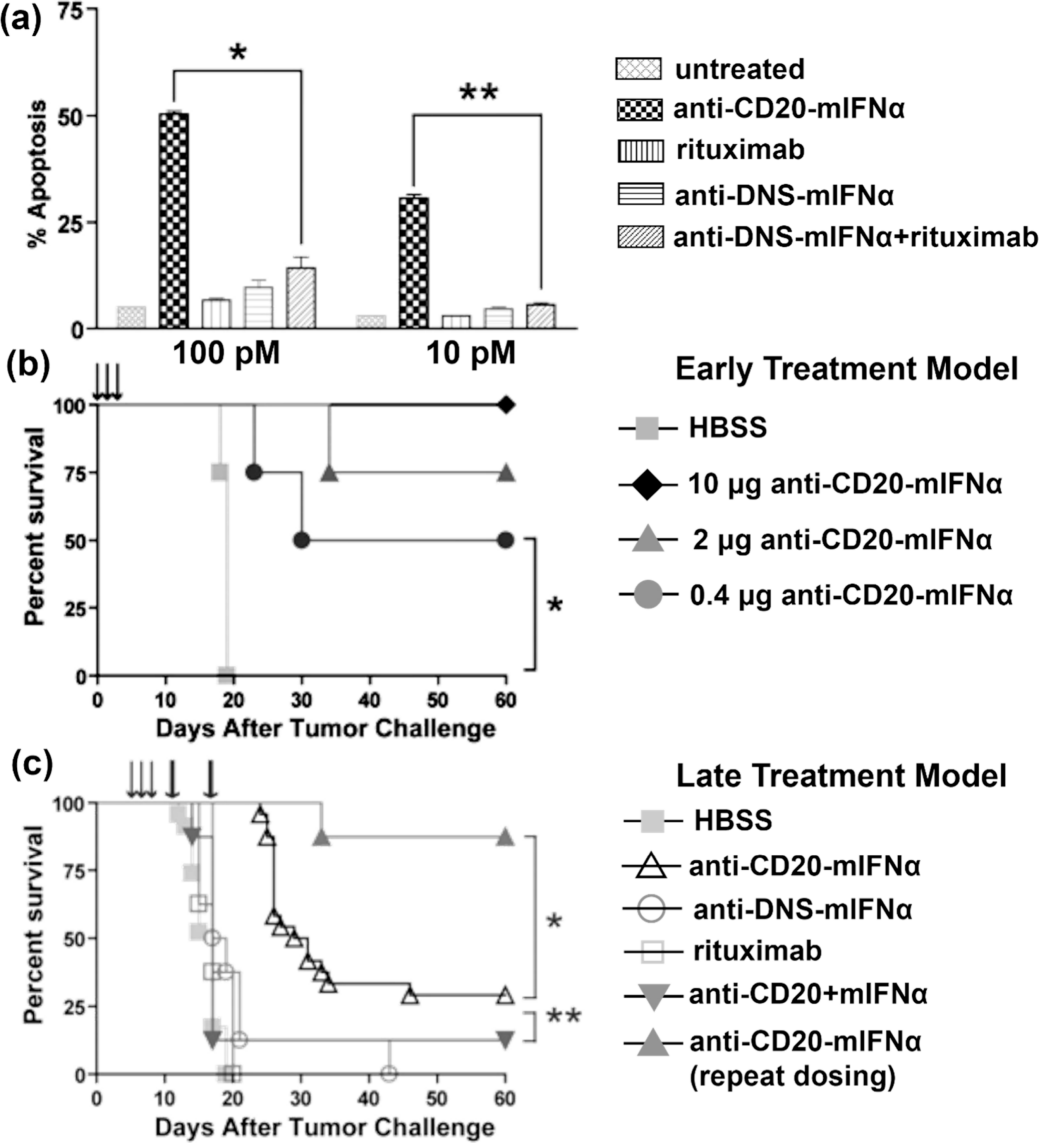
Anticancer potential of anti-CD20-mIFN. (a) Anti-CD20-mIFN potently induces apoptosis of 38C13-huCD20 cells in a dose-dependent manner. (b and c) Anti-CD20-mIFN treatment can prevent tumor establishment and eradicate established, rituximab insensitive human CD20 B-cell lymphoma. Reproduced with permission from ref (103). Copyright 2010 The American Society of Hematology.

Interferon α (IFN-α) is involved in the treatment of B-cell non-Hodgkin lymphomas. However, the systemic administration of IFN-α often results in severe toxicities. Xuan et al. fused IFN-α onto anti-CD20 antibodies (anti-CD20-mIFN),^103^ which specifically binds to CD20 on the surface of cancer cells. Flow cytometry data confirmed that the binding affinity for CD20 remained unchanged after fusion with IFN-α. *In vitro* apoptosis assays demonstrated a 105-fold increase in antiproliferative activity against the CD20-expressing murine B cell lymphoma 38C13 cell line when using the fusion protein compared to commercially available rituximab and a nonspecific antibody fused with IFN-α (anti-DNS-mIFNα) (Figure 8a). The fusion proteins also showed excellent therapeutic efficacy in preventing the establishment of 38C13 tumors and in eradicating established tumors without any apparent toxicity (Figure 8b,c).

It is worth noting that antibodies alone do not need to possess intrinsic antitumor activity after fusion. However, if antitumor activity is retained postfusion, the therapeutic efficacy of the fused cytokines is expected to surpass that of native cytokines.

Antibody-conjugated or -fused cytokines have a high manufacturing cost due to the complex production process. They have also shown side effects, including injection site reaction.^104^ The conjugated or fused antibody dramatically increases the size of cytokines, reducing their rate of diffusion in tissues. The binding between the antibody and antigens further impairs the tissue penetration of the modified cytokines in, for example, solid tumors in cancer treatment.^105^

### 3.6 Other Methods

Several other methods have been developed for enhancing protein delivery. One well-known technique is glycosylation, which involves the introduction of C-glycans or N-glycans at specific sites in therapeutic proteins.^106^ This is achieved by mutation in recombinant proteins to create glycosylation sites.^106^ The added glycans enhance the thermal stability and solubility of proteins, prevent the proteins from being proteolyzed or aggregated, and prolong their circulation half-life. Specifically, glycans at designated sites can interfere with receptors in immune cells, thereby reducing receptor-mediated clearance. The introduction of glycans increases the molecular weight of the fused proteins, and their hydrophilic nature attracts additional water molecules.^107,108^ In 2010, Flintegaard et al. reported that N-glycosylation prolongs the circulation half-life of growth hormone (GH).^109^ The introduction of three glycans increased the molecular weight of GH from 22 to 31 kDa. The sialic acid units in these glycans were hydrophilic, leading to a further increase in hydrodynamic size. GH with N-glycans demonstrated a lower susceptibility against receptor-mediated clearance. Furthermore, the negatively charged sialic acid residues within glycans provided electrostatic repulsion from membranes in the glomerular filter.^110^ Together, these factors increased the GH circulation half-life from 0.23 to 5.6 h in rats. In addition, glycosylation has also been shown to extend the circulation half-life of proteins such as erythropoietin,^111^ follicle-stimulating hormone (FSH),^112^ and interferon-α2.^113^ However, glycosylation is a highly complex process that typically produces heterogeneous glycan structures, complicating quality control and chemical and biological assays.^114^

Along with endogenous proteins. such as IgG and albumin, red blood cells (RBCs) also show a long circulation half-life in the human body. This longevity is attributed to the zwitterionic lipids, CD47, a marker of “self”, and complement regulators on the RBC membrane.^115^ The CD47 signals “self” when interacting with signal regulatory protein-α (SIRPα) on macrophages, while complement activation is regulated by complement regulators.^115^ In addition to their extended circulation half-life, RBCs are biocompatible and biodegradable, making them excellent candidates as drug carriers. Various protein drugs, including acetaldehyde and alcohol dehydrogenase, as well as erythropoietin, have been encapsulated in RBCs to extend their circulation half-lives.^116,117^l-Asparaginase (ASNase) is a protein drug used to treat acute lymphoblastic leukemia. It has a short circulation half-life, requiring frequent injections, and often leads to hypersensitivity reactions in patients.^118^ A conventional method to protect ASNase from rapid clearance involves hypoosmotic rupture and resealing of RBCs after encapsulation.^119^ In 2009, Kwon et al. developed a low-molecular weight protamine (LMWP) conjugated to l-asparaginase that can translocate into RBCs without disrupting the cell membrane.^120^ It has been reported that this approach increased the circulation half-life of l-asparaginase from 2.4 days with conventional methods to 4.5 days. Nevertheless, a major limitation in the use of RBCs as therapeutic protein carriers is that the process of loading proteins drugs into RBCs can disrupt the cell membranes, causing irreversible changes in the physicochemical properties of RBCs.^121^ This damage expedites their removal *in vivo* by the reticuloendothelial system (RES).^117^

Nanoparticles (NPs) have broad biomedical applications. It can shield encapsulated proteins from the immune system. In protein delivery, NPs offer several advantages, including protecting proteins from premature degradation or denaturation in biological environments, extending the systemic circulation half-life of proteins with poor pharmacokinetic properties, and enabling controlled and tunable release to maintain drug concentrations within the therapeutic range. NPs also mediate targeted delivery to diseased tissues, cells, and intracellular compartments, enhancing the safety and efficacy of biologic therapeutics.^122^ An interesting work published recently developed a novel NP based on poly(disulfide)s,^123^ which could encapsulate a variety of proteins through noncovalent interaction. This novel NP achieved intracellular delivery of proteins through the thiol-mediated uptake pathway, overcoming limitations of classic endocytosis. The group also demonstrated great efficacy in treating HeLa tumor-bearing mice using their NP-encapsulating saporin. Despite their advantages, NPs also have notable drawbacks, such as protein denaturation during the encapsulation process, a low loading efficiency, burst release-caused side effects, and challenges in manufacturing and administration.^122,124^

## 4 Conclusions and Perspectives

Therapeutic proteins have become mainstream drugs. The kidney and liver are the two primary organs in the clearance of these drugs, with clearance rates largely determined by the size and binding affinity of proteins for immune cells. Most research has focused on increasing the hydrodynamic size of protein therapeutics or minimizing their binding to immune cells. PEGylation has emerged as a widely accepted method for prolonging protein circulation in the blood as conjugated PEG chains can stabilize proteins, reduce both renal clearance and receptor-mediated clearance, and protect proteins from proteolysis. Advances in conjugation techniques have allowed precise control of the number of attached PEG chains, making the circulation profile controllable and reducing the manufacturing cost. However, concerns regarding PEGylation have arisen, particularly related to its nondegradability, decreased bioactivity of the PEGylated proteins, and the induction of anti-PEG antibodies through repeated injections of PEGylated immunogenic proteins. Complex purification steps may also be required to generate homogeneous PEGylated products.

XTENylation shares some similarities with PEGylation in extending the circulation half-life of protein therapeutics. Unlike PEG, XTEN is biodegradable and highly compatible and does not induce anti-XTEN antibodies in reported studies. XTEN can also be fused at desired sites on protein drugs, minimizing the effect of bulky XTEN on their bioactivity. Furthermore, the circulation profile can be controlled with the desired number and length of XTEN chains. However, both PEG and XTEN can interfere with the interaction between delivered proteins and their targets unless the targets have a low molecular weight. In certain applications, the shedding of these polymers from proteins in response to the tissue microenvironment may enhance the efficacy. Other disadvantages of XTENylation include a high manufacturing cost, reduced rates of diffusion in tissues, and an altered biodistribution.

Fusing the Fc region of IgG or attaching albumin to therapeutic peptides or proteins has demonstrated prolonged circulation via the FcRn-mediated recycling mechanism. These modifications can be selectively introduced at specific sites within proteins and are expected to confer extended blood circulation akin to that of IgG or albumin. However, experimental data have consistently shown that the actual circulation half-lives of these modified proteins are shorter than expected, likely due to variations in binding affinities for FcRn.^10,99^ Albumin attachment has several advantages over Fc fusion in protein and peptide delivery.

Antibody-conjugated or -fused cytokines have been used for cancer therapy. The modification dramatically enhances the selectivity of cytokines but at the cost of reduced bioactivity and rates of protein diffusion in tissues.

Other potential strategies to improve circulation half-life of protein drugs include fusing with other plasma proteins,^125^ employing glycosylation and polysialylation techniques,^126^ and encapsulating proteins within RBCs or NPs.^127−129^ Glycosylation can enhance the stability and solubility of proteins but also increases their complexity and heterogeneity. RBCs as protein carriers have good biocompatibility and degradability. However, the process of loading a drug into RBCs may damage cells and expedite their clearance by RES. The high manufacturing and storage costs also limit its application. Placing therapeutics on the surfaces of RBCs or other cells instead of inside of the cells may reduce the degree of damage to the cells, slow RES clearance, and provide convenience in manufacturing and application by direct attachment to the cells within the bloodstream.^130−133^ Using NPs as protein carriers offers several advantages, including reduced degradation, targeted delivery, and controlled release. However, significant drawbacks remain, ranging from protein denaturation to low loading yields.

Looking to the future, several promising directions could further improve the circulation half-life and efficacy of protein therapeutics. Recent advancements in polymer science have prompted investigations into alternative substitutes for PEG. Potential alternatives include zwitterionic polymers, polysulfoxides, poly(2-methyl-2-oxazoline), and poly(vinyl-pyrrolidone).^134−139^ Zwitterionic polymers are among the most promising candidates.^140,141^ These polymers feature equal amounts of positively and negatively charged groups, which confer super-hydrophilicity and attract water molecules to prevent nonspecific protein adsorption. Zwitterionic polymers have been shown to be less immunogenic than PEG, positioning them as a viable option for extending blood circulation and shielding therapeutics from immune responses. However, considerable work remains to establish the superiority of zwitterionic polymers over PEG. Although progress has been made in these areas, many systems remain underdeveloped and need to be substantially refined. In summary, techniques for prolonged protein circulation have their own pros and cons (Table 5). A suitable technique may be selected, depending on the required biodistribution, size of targeted molecules, and physicochemical properties of therapeutic proteins. Future protein delivery strategies should be advanced to enable well-controlled biodistribution and pharmacokinetics, efficient bioactivity, high biocompatibility, low immunogenicity, minimal side effects, and cost-effective manufacturing. Achieving this goal requires the integration of materials science, protein engineering, and translational research.

**Table 5:** Summary of the Pros and Cons of Commonly Used Strategies for Protein Delivery

strategy	advantages	disadvantages
PEGylation	enhanced protein stability	decreased bioactivity
	cost efficient manufacture	anti-PEG antibodies may reduce therapeutic efficacy and cause hypersensitivity
	controlled circulation profile	nondegradability
XTENylation	high biocompatibility and degradability	high manufacturing cost
	site-specific modification	reduced rates of diffusion in tissues and altered biodistribution
	controlled circulation profile	decreased bioactivity
Fc fusion	ultralong circulation	reduced rates of diffusion in tissues
	high biocompatibility	Fc-medicated effector functions
	site-specific modification	complex manufacturing
		uncontrolled pharmacokinetics
		protein destabilization
albumin attachment	high biocompatibility	binding to nontargeted cells via albumin receptors
	site-specific modification	reduced rates of diffusion in tissues
antibody-conjugated cytokine	high selectivity	side effects
		reduced rates of diffusion in tissues and altered biodistribution
glycosylation	enhanced protein stability and solubility	complexity and heterogeneity
encapsulation in RBCs	biocompatibility and biodegradability	disruption of the cell membrane
		clearance by the RES
		complexity in manufacturing and cost
nanoparticles	reduced protein degradation	protein denaturation
	controlled release	low loading efficiency
	improved targeted delivery, safety, and efficacy	burst release leading to side effects
		challenges in manufacturing and administration
